# Anthrax in Red Deer (*Cervus elaphus*), Italy

**DOI:** 10.3201/eid1307.061465

**Published:** 2007-07

**Authors:** Antonio Fasanella, Lucia Palazzo, Antonio Petrella, Vincenzo Quaranta, Bruno Romanelli, Giuliano Garofolo

**Affiliations:** * Istituto Zooprofilattico Sperimentale of Puglia and Basilicata, Foggia, Italy

**Keywords:** Red deer, anthrax, outbreaks, Cervus elaphus, Italy, letter

**To the Editor:** Anthrax is hypoendemic in Italy; a few outbreaks occurred yearly involving unvaccinated herbivores on pastures in central and southern regions and the major islands. Multiple-locus variable-number tandem-repeat analysis (MLVA) with 8 variable-number tandem repeats (VNTRs) of Italian isolates of *Bacillus anthracis* has identified 9 genotypes belonging to cluster A1a (*1*). An isolate of cluster A3 has been identified recently in Sardinia, which suggests that such a strain could have been introduced into Italy from another country (*1*).

A total of 37 anthrax outbreaks occurred in a 41-day period from August 28 to October 3, 2004, in a restricted area of Pollino National Park (Basilicata region in southern Italy) and resulted in the deaths of 124 domestic or wild animals. Two suspected cases of cutaneous anthrax in humans were recorded. Pollino National Park contains several species of feral animals. Since 1990, there has been a program for reintroduction of red deer (*Cervus elaphus*) into this park from Tuscany, Italy, and Carinthia, Austria. The animals are kept in quarantine in a corral by the veterinary services of the park and given an electronic tag before their release. At the time of the anthrax outbreaks, the red deer population of the park was 45, of which 10 were living in the corral. These outbreaks killed 8 deer (4 free-ranging and 4 confined animals).

Each carcass was examined by the veterinary officer, who collected clinical samples that were examined for *B*. *anthracis* by using standard procedures of the Istituto Zooprofilattico Sperimentale of Puglia and Basilicata. DNA from the suspected colonies was analyzed by PCR with primers specific for *B*. *anthracis* (*2*) and subsequent genotyping by using MLVA with 8 VNTRs (*3*). All *B*. *anthracis* isolates belonged to cluster A1a, genotype 1 (A. Fasanella, unpub. data). This genotype was also identified in subsequent outbreaks that involved farm animals in the same area and resulted in the deaths of 116 domestic animals, including 81 cattle, 15 sheep, 9 goats, and 11 horses. Red deer showed the highest mortality rate during these outbreaks (Table). An ELISA (*4*) performed with 27 serum samples obtained from deer in the park detected low levels of antibodies to *B*. *anthracis* in 22% of the examined animals. This seroprevalence is consistent with levels found in unvaccinated livestock reared in areas endemic for anthrax (A. Fasanella, unpub. data).

**Table:** Mortality rates during anthrax outbreaks, Italy, 2004

Animal	Population of area	No. (%) dead animals
Cattle	≈7,000	81 (≈1.15)
Sheep	≈20,000	15 (≈0.075)
Goats	≈13,000	9 (≈0.069)
Horses	≈600	11 (≈1.83)
Red deer	45	8 (≈17.77)

A vaccination program was then instituted for farm animals, but the deer population in the park was excluded because no experimental data were available on the safety and efficacy of Carbosap vaccine (Istituto Zooprofilattico Sperimentale of Puglia and Basilicata, Foggia, Italy) in wild ruminants. Extensive vaccination limited the outbreaks in livestock and red deer, which probably prevented further spread of infection from farm animals to free-ranging deer.

These anthrax outbreaks in southern Italy suggested that red deer are highly susceptible to infection with *B*. *anthracis* and that the mortality rate in these deer could be even higher than that observed in domestic animals. Although epidemiologic data are limited and need to be supported experimentally by assessment of the 50% lethal dose of *B*. *anthracis* in red deer, the ecologic effect on deer populations in parks should not be underestimated. Moreover, concerns for public health may arise in parks in disease-endemic areas, where susceptible wild animals could represent an amplification factor for *B*. *anthracis* spores, which increases the probability of outbreaks in domestic animals and in humans living near, working in, or visiting the parks. This article stresses the need for evaluating the safety and efficacy of *B*. *anthracis* vaccines in deer and for including wild ruminants in the anthrax prophylaxis programs.

## References

[1] Fasanella A, van Ert M, Altamura SA, Garofolo G, Buonavoglia C, Leori G, Molecular diversity of *Bacillus anthracis* isolates in Italy. J Clin Microbiol. 2005;43:3398–401. 10.1128/JCM.43.7.3398-3401.2005 16000465 PMC1169099

[2] Fasanella A, Losito S, Trotta T, Adone R, Massa S, Ciuchini F, Detection of anthrax vaccine virulence factors by polymerase chain reaction. Vaccine. 2001;19:4214–8. 10.1016/S0264-410X(01)00159-1 11457547

[3] Keim P, Price LB, Klevytska AM, Smith KL, Schupp JM, Okinaka R, Multiple-locus variable-number tandem repeat analysis reveals genetic relationships within *Bacillus anthracis.* J Bacteriol. 2000;182:2928–36. 10.1128/JB.182.10.2928-2936.2000 10781564 PMC102004

[4] Fasanella A, Fabiano MP, Garofolo G, Losito S. Preliminary studies on recombinant protective antigen (rPA) vaccine against anthrax for veterinary use. In: Abstracts of the International Veterinary Vaccines and Diagnostic Conference. Oslo, Norway; Jun 25–29, 2006. Oslo: University of Oslo; 2006. p. 69.

